# Publisher Correction: Inference of RNA decay rate from transcriptional profiling highlights the regulatory programs of Alzheimer’s disease

**DOI:** 10.1038/s41467-018-07153-6

**Published:** 2018-10-31

**Authors:** Rached Alkallas, Lisa Fish, Hani Goodarzi, Hamed S. Najafabadi

**Affiliations:** 1 Department of Human Genetics, McGill University, Montreal, QC, H3A 0C7 Canada; 2 McGill University and Genome Quebec Innovation Centre, Montreal, QC, H3A 0G1 Canada; 3 Department of Biochemistry and Biophysics, University of California, San Francisco, CA 94158 USA; 4 Department of Urology, University of California, San Francisco, CA 94158 USA; 5 Helen Diller Family Comprehensive Cancer Center, University of California, San Francisco, CA 94158 USA

Correction to: *Nature Communications*; 10.1038/s41467-017-00867-z; published online: 13 Oct 2017.

The original version of this Article contained an error in Figure 3, where panel d was inadvertently replaced with a duplicate of panel c during typesetting. Also, the legend of Figure 5f incorrectly read ‘310 AD patients (blue dots, *r* = –0.4) and 157 non-demented individuals (green dots, *r* = –0.1)’, and should have read ‘310 AD patients (blue dots, *r* = –0.1) and 157 non-demented individuals (green dots, *r* = –0.4)’. Both of these errors have now been corrected in both the PDF and HTML versions of the Article.

