# Supplementary material for: Inference of RNA decay rate from transcriptional profiling highlights the regulatory programs of Alzheimer’s disease
Source: Nat Commun. 2017 Oct 13;8:909. doi: 10.1038/s41467-017-00867-z (PMC5714957; doi:10.1038/s41467-017-00867-z)
Supplement: Supplementary file 1 — Supplementary Information [file 41467_2017_867_MOESM1_ESM.pdf]

Description of Supplementary Files

File Name: Supplementary Information
Description: Supplementary Figures, Supplementary Table and Supplementary References

File Name: Peer Review File

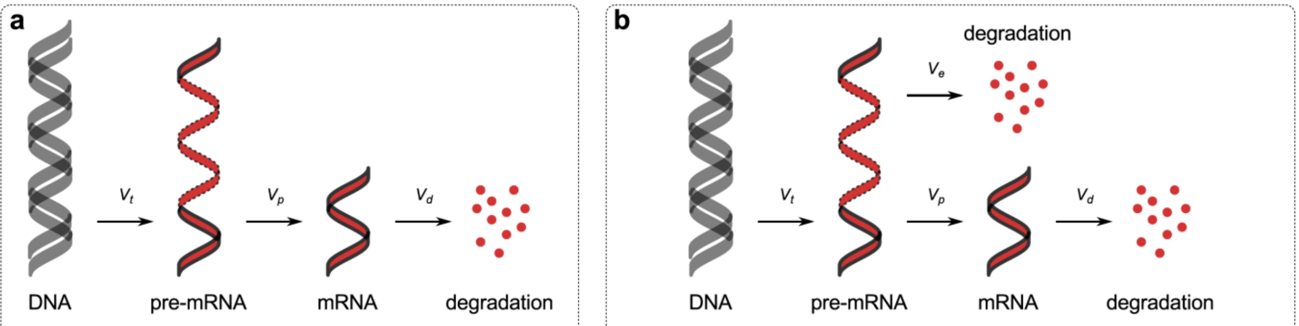

a

$$V_t = V_p = V_d$$

$$V_t = V_p = \frac{V_{p,\max}[pre-mRNA]}{K_p + [pre-mRNA]} \quad (Eq.\ 1)$$

$$V_{p,\max}[pre-mRNA] = V_t K_p + V_t[pre-mRNA]$$

$$[pre-mRNA] = \frac{K_p}{\frac{V_{p,\max}}{V_t} - 1}$$

$$\frac{[pre-mRNA]'}{[pre-mRNA]} = \frac{\frac{V_{p,\max}}{V_t} - 1}{\frac{V_{p,\max}}{V'_t} - 1} \quad (Eq.\ 2)$$

$$V_t = V_d = k_d[mRNA]$$

$$[mRNA] = \frac{V_t}{k_d}$$

$$\frac{[mRNA]'}{[mRNA]} = \frac{V'_t k_d}{V_t k'_d} \quad (Eq.\ 3)$$

$$(Eq.\ 2\ and\ 3) \Rightarrow \frac{\frac{[mRNA]'}{[mRNA]}}{\frac{[pre-mRNA]'}{[pre-mRNA]}} = \frac{V'_t k_d}{V_t k'_d} \times \frac{\frac{V_{p,\max}}{V'_t} - 1}{\frac{V_{p,\max}}{V_t} - 1} = \frac{k_d}{k'_d} \times \frac{V_{p,\max} - V'_t}{V_{p,\max} - V_t}$$

$$\Rightarrow \Delta e - \Delta i = \log\frac{k_d}{k'_d} + \log\frac{V_{p,\max} - V'_t}{V_{p,\max} - V_t} \quad (Eq.\ 4)$$

$$(Eq.\ 1\ and\ 4) \Rightarrow \Delta e - \Delta i = \log\frac{k_d}{k'_d} + \log\frac{V_{p,\max} - \frac{V_{p,\max}[pre-mRNA]'}{K_p + [pre-mRNA]'}}{V_{p,\max} - \frac{V_{p,\max}[pre-mRNA]}{K_p + [pre-mRNA]}}$$

$$\Rightarrow \Delta e - \Delta i = \log\frac{k_d}{k'_d} + \log\frac{K_p + [pre-mRNA]}{K_p + [pre-mRNA]'}$$

b

$$V_t = V_p + V_e = V_d + V_e \Rightarrow V_t - V_e = V_p = V_d$$

$$V_t - V_e = V_p = \frac{V_{p,\max}[pre-mRNA]}{K_p + [pre-mRNA]} \quad (Eq.\ 1)$$

$$V_{p,\max}[pre-mRNA] = (V_t - V_e)K_p + (V_t - V_e)[pre-mRNA]$$

$$[pre-mRNA] = \frac{K_p}{\frac{V_{p,\max}}{V_t - V_e} - 1}$$

$$\frac{[pre-mRNA]'}{[pre-mRNA]} = \frac{\frac{V_{p,\max}}{V_t - V_e} - 1}{\frac{V_{p,\max}}{V'_t - V'_e} - 1} \quad (Eq.\ 2)$$

$$V_t - V_e = V_d = k_d[mRNA]$$

$$[mRNA] = \frac{V_t - V_e}{k_d}$$

$$\frac{[mRNA]'}{[mRNA]} = \frac{(V'_t - V'_e)k_d}{(V_t - V_e)k'_d} \quad (Eq.\ 3)$$

$$(Eq.\ 2\ and\ 3) \Rightarrow \frac{\frac{[mRNA]'}{[mRNA]}}{\frac{[pre-mRNA]'}{[pre-mRNA]}} = \frac{(V'_t - V'_e)k_d}{(V_t - V_e)k'_d} \times \frac{\frac{V_{p,\max}}{V'_t - V'_e} - 1}{\frac{V_{p,\max}}{V_t - V_e} - 1} = \frac{k_d}{k'_d} \times \frac{V_{p,\max} - (V'_t - V'_e)}{V_{p,\max} - (V_t - V_e)}$$

$$\Rightarrow \Delta e - \Delta i = \log\frac{k_d}{k'_d} + \log\frac{V_{p,\max} - (V'_t - V'_e)}{V_{p,\max} - (V_t - V_e)} \quad (Eq.\ 4)$$

$$(Eq.\ 1\ and\ 4) \Rightarrow \Delta e - \Delta i = \log\frac{k_d}{k'_d} + \log\frac{V_{p,\max} - \frac{V_{p,\max}[pre-mRNA]'}{K_p + [pre-mRNA]'}}{V_{p,\max} - \frac{V_{p,\max}[pre-mRNA]}{K_p + [pre-mRNA]}}$$

$$\Rightarrow \Delta e - \Delta i = \log\frac{k_d}{k'_d} + \log\frac{K_p + [pre-mRNA]}{K_p + [pre-mRNA]'}$$

Supplementary Figure 1. Modeling Δ*exon*–Δ*intron* as a function of mRNA degradation rate and pre-mRNA concentration. (**a**) A simple model of mRNA metabolism, consisting of transcription at the rate of V_t, pre-mRNA processing at the rate of V_p, and mRNA degradation at the rate of V_d. Solving the rate equations with the assumption of steady state (which leads to V_t=V_p=V_d) as well as invariability of $V_{p,max}$ (the maximum rate of pre-mRNA processing at saturation) and K_p (the Michaelis-Menten constant of pre-mRNA processing), results in the equation at the bottom, which connects Δ*exon*–Δ*intron* to the change in mRNA degradation and pre-mRNA concentration. (**b**) A more complicated model, in which nuclear exosomal degradation of pre-mRNA [1] is also taken into account, leads to the same equation as in (**a**).

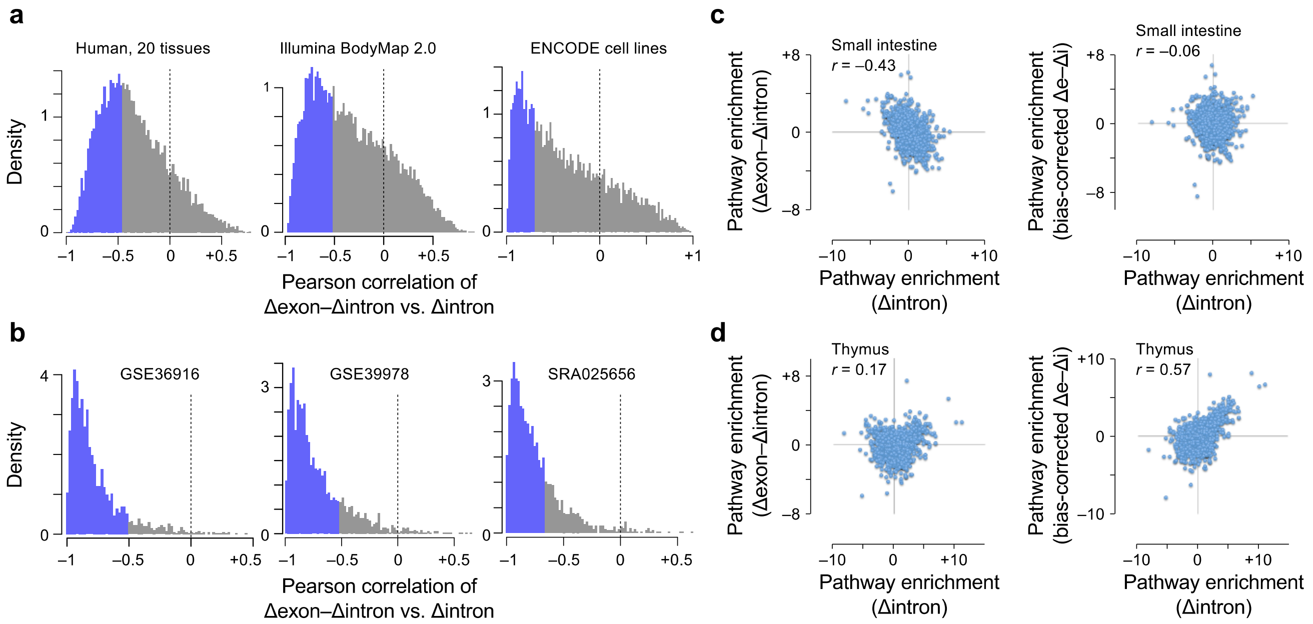

Supplementary Figure 2. Δ*exon*–Δ*intron* is a biased estimate of mRNA stability. (**a**) Histogram of per-gene Pearson correlation coefficients for Δ*exon*–Δ*intron* vs. Δ*intron* for three different human RNA-seq datasets. The median Pearson correlations, from left to right, are –0.40, –0.38, and –0.47, respectively ($P < 10^{-300}$ in all cases, two-sided t-test). The blue fraction of each histogram corresponds to the genes with significant negative Pearson correlations (FDR<0.05). (**b**) Same as in (**a**), for three different mouse RNA-seq datasets. The GEO or SRA accession number of each dataset is indicated on top. The median Pearson correlations, from left to right, are –0.84, –0.80, and –0.81, respectively ($P < 10^{-100}$ in all cases, two-sided t-test). (**c**) Pathway enrichment analysis of Δ*exon*–Δ*intron* results in an apparent anti-correlation between transcriptional and post-transcriptional regulation of pathways in small intestine. In each scatter plot, each dot represents one REACTOME [2] pathway. The x-axes correspond to the Mann-Whitney U test z-score of the pathway genes with respect to Δ*intron*, and the y-axes correspond to the Mann-Whitney U test z-score with respect to Δ*exon*–Δ*intron* (left) or the bias-corrected measure introduced in this work (right). (**d**) Same as in (**c**) for thymus. Note the increased correlation between transcriptional and post-transcriptional change in pathways after correcting for bias.

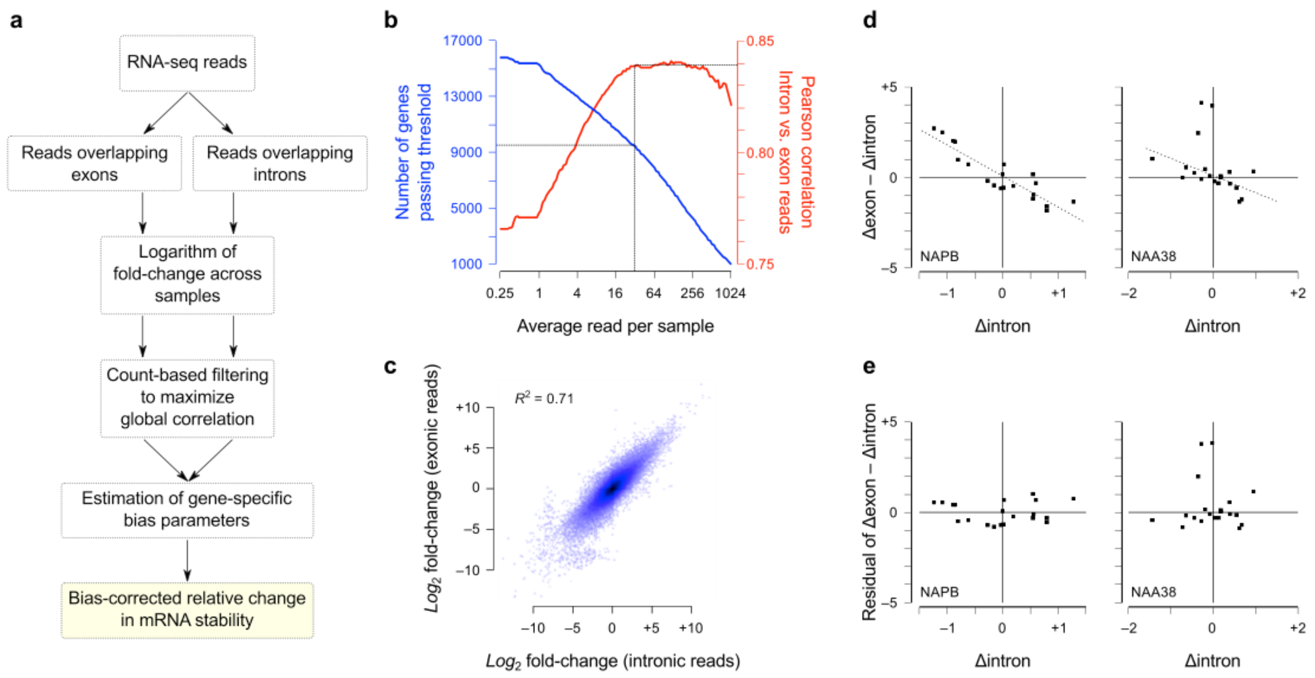

Supplementary Figure 3. Obtaining an unbiased estimate of differential mRNA stability. (**a**) A simplified diagram of REMBRANDTS (REMoving Bias from Rna-seq ANalysis of Differential Transcript Stability, https://github.com/csglab/REMBRANDTS). REMBRANDTS first estimates a minimum count of exonic and intronic reads that would still allow us to reliably measure Δ*exon* and Δ*intron*. For this, we take advantage of the correlation that we expect to see between Δ*exon* and Δ*intron* (Methods, and ref [3]). Low read counts result in noisy estimates of these two measures, leading to a reduction in their correlation. REMBRANDTS chooses a minimum read count cutoff that results in a near maximum correlation between Δ*exon* and Δ*intron*. Next, it estimates the bias function for each gene by linear regression of Δ*exon*–Δ*intron* vs. Δ*intron*, and takes the residual of regression as an unbiased alternative to Δ*exon*–Δ*intron*. (**b**) Finding read count cutoff for genes based on the correlation between Δ*exon* and Δ*intron* in an RNA-seq dataset of 20 human tissues [4]. The x-axis represents the possible read count cutoffs. The red curve denotes the correlation between Δ*exon* and Δ*intron* at each cutoff, and the blue curve denotes the number of genes that pass each cutoff. (**c**) The scatterplot of Δ*exon* vs. Δ*intron* for the set of genes that pass the intron/exon read count cutoff. Each data point represents one gene in one of the 20 human tissues we examined. (**d**) The scatterplots of Δ*exon*–Δ*intron* vs. Δ*intron* for two example genes are shown. Each data point represents one tissue. The dotted line represents the least-square linear fit, which serves as an estimate of the bias of Δ*exon*–Δ*intron*. (**e**) The scatterplot of Δ*exon*–Δ*intron* vs. Δ*intron* for the same two genes after subtracting the estimated bias.

a

$$V_t = V_p = V_d$$

$$V_t = V_p = \frac{V_{p,\max}[pre-mRNA]}{K_p + [pre-mRNA]} \quad (Eq.\,1)$$

$$V_{p,\max}[pre-mRNA] = V_t K_p + V_t[pre-mRNA]$$

$$[pre-mRNA] = \frac{K_p}{\frac{V_{p,\max}}{V_t} - 1}$$

$$\frac{[pre-mRNA]'}{[pre-mRNA]} = \frac{K'_p}{K_p} \times \frac{\frac{V_{p,\max}}{V_t} - 1}{\frac{V'_{p,\max}}{V'_t} - 1} \quad (Eq.\,2)$$

$$V_t = V_d = k_d[mRNA]$$

$$[mRNA] = \frac{V_t}{k_d}$$

$$\frac{[mRNA]'}{[mRNA]} = \frac{V'_t k_d}{V_t k'_d} \quad (Eq.\,3)$$

$$(Eq.\,2\ and\ 3) \Rightarrow \frac{\frac{[mRNA]'}{[mRNA]}}{\frac{[pre-mRNA]'}{[pre-mRNA]}} = \frac{V'_t k_d}{V_t k'_d} \times \frac{K_p}{K'_p} \times \frac{\frac{V'_{p,\max}}{V'_t} - 1}{\frac{V_{p,\max}}{V_t} - 1} = \frac{k_d}{k'_d} \times \frac{K_p}{K'_p} \times \frac{V'_{p,\max} - V'_t}{V_{p,\max} - V_t}$$

$$\Rightarrow \Delta e - \Delta i = \log\frac{k_d}{k'_d} + \log\frac{K_p}{K'_p} + \log\frac{V'_{p,\max} - V'_t}{V_{p,\max} - V_t} \quad (Eq.\,4)$$

$$(Eq.\,1\ and\ 4) \Rightarrow \Delta e - \Delta i = \log\frac{k_d}{k'_d} + \log\frac{K_p}{K'_p} + \log\frac{V'_{p,\max}}{V_{p,\max}} + \log\frac{\frac{K'_p}{K'_p + [pre-mRNA]'}}{\frac{K_p}{K_p + [pre-mRNA]}}$$

$$\Rightarrow \Delta e - \Delta i = \log\frac{k_d}{k'_d} + \log\frac{V'_{p,\max}}{V_{p,\max}} + \log\frac{K_p + [pre-mRNA]}{K'_p + [pre-mRNA]'}$$

b

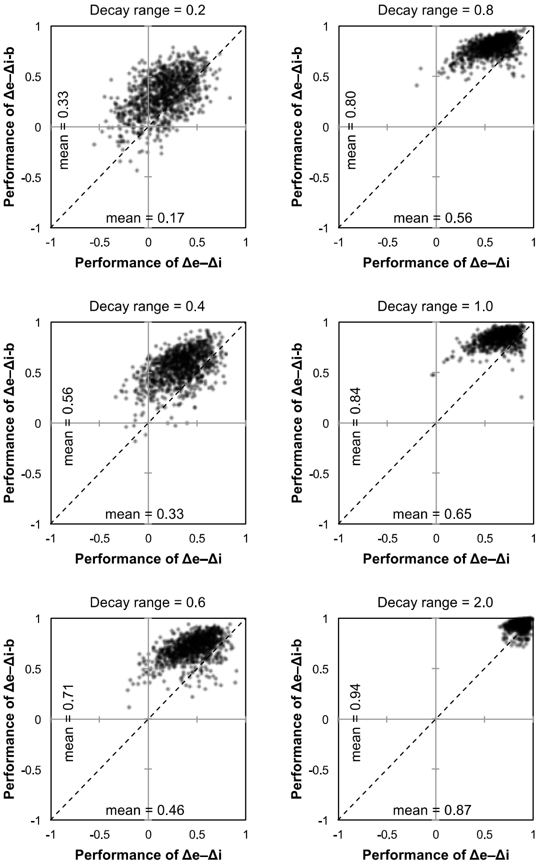

Supplementary Figure 4. Modeling Δ*exon*–Δ*intron* when $V_{p,max}$ and K_p are not constant across samples. (**a**) Kinetic rate equations of mRNA metabolism without the assumption of invariability of $V_{p,max}$ (the maximum rate of pre-mRNA processing at saturation) and K_p (the Michaelis-Menten constant of pre-mRNA processing). Note that when $\Delta\log V_{p,max} << \Delta\log k_d$ and $\Delta K_p << \Delta$[pre-mRNA], the equation at the bottom reduces to the equation presented in **Supplementary Figure 1a**. (**b**) REMBRANDTS assumes that K_p and $V_{p,max}$ are invariable across samples, and therefore the bias only depends on the change in transcription rate (**Supplementary Figure 1a**). We simulated one thousand genes across 20 tissues, for which the rates of transcription and mRNA decay as well as the $V_{p,max}$ and K_p vary across 20 tissues. Log_2 of ratio of transcription rate (relative to average across tissues) for each gene in each tissue was randomly sampled from a normal distribution with a mean of zero and standard deviation of 2.0. The log_2 of ratio of K_p vs. pre-mRNA abundance, as well as log_2 ratio of $V_{p,max}$ vs. average $V_{p,max}$ across tissues were sampled from a uniform distribution in the range [–2,+2]. Then we ran six simulations with varying ranges of mRNA decay rate: log_2 of ratio of mRNA decay rate (relative to average across tissues) was sampled from a normal distribution with a mean of zero and standard deviation of 0.2, 0.4, 0.6, 0.8, 1.0, or 2.0. In each simulation, the change in log_2 of pre-mRNA (Δ*intron*) and mature mRNA (Δ*exon*) for each gene in each tissue was calculated based on the kinetic rate equations of the model in (**a**) using the sampled values of transcription rate, decay rate, $V_{p,max}$ and K_p. Then, for each gene, the uncorrected Δ*exon*–Δ*intron* in each tissue was calculated. Also, Δ*exon* and Δ*intron* across the 20 simulated tissues for each gene was used to estimate and remove the bias as in **Figure 2a** and **Supplementary Figure 3d**. The uncorrected and bias-corrected Δ*exon*–Δ*intron* was then compared to the original simulated mRNA decay rates. In each panel, each data point represents one gene, the x-axis represents the Pearson correlation of uncorrected Δ*exon*–Δ*intron* vs. the simulated mRNA decay rates, and the y-axis represents the Pearson correlation of bias-corrected Δ*exon*–Δ*intron* vs. the simulated mRNA decay rates. In all six cases, bias-corrected Δ*exon*–Δ*intron* overall outperforms the uncorrected measures (two-sided paired t-test $P<10^{-100}$).

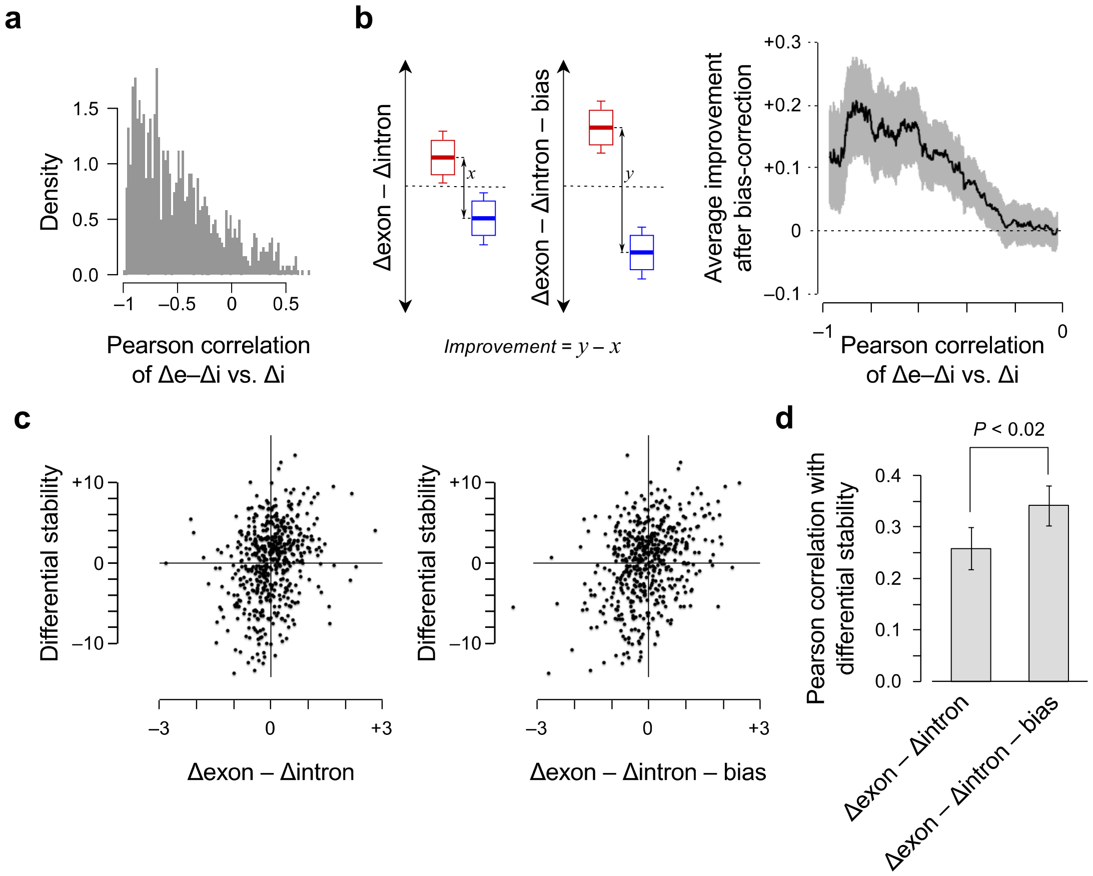

Supplementary Figure 5. Comparison of uncorrected and corrected estimates of mRNA stability. (**a**) Histogram of per-gene Pearson correlation coefficients for Δ*exon*–Δ*intron* vs. Δ*intron*, based on RNA-seq data from ref [5]. Only the genes with previously reported differential stability between MDA-parental and MDA-LM2 cells[6] are included. (**b**) Improvement in inferring differential mRNA stability after bias correction for genes with varying degrees of bias, based on analysis of RNA-seq data for MDA-parental and MDA-LM2 cells[5,6]. Left: Schematic of how improvement is defined (red: stabilized genes, blue: destabilized genes, similar to Figure 2). Right: Improvement as a function of bias. Genes were sorted based on their Pearson correlation of Δ*exon*–Δ*intron* vs. Δ*intron*, and average improvement was calculated for sliding windows of 500 genes. The x-axis corresponds to the average Pearson correlation for each sliding window. The shaded area represents standard error of mean. (**b**) Scatterplot of uncorrected (Δ*exon*–Δ*intron*, top) or corrected (Δ*exon*–Δ*intron*–*bias*, bottom) estimates vs. differential mRNA half-life between mouse embryonic stem cells (ESCs) and ESCs differentiated to terminal neurons. Exonic and intronic read counts were taken from ref [3] (original RNA-seq data from GEO dataset GSE33252), and were processed using REMBRANDTS (see **Supplementary Figure 3**). In order to estimate the gene-specific bias terms, we also included three other sets of exonic and intronic read counts from ref [3] (original RNA-seq data from GEO datasets GSE36916 and GSE39978 and SRA dataset SRA025656). The mRNA half-life measures were also taken from ref [3] (based on data from GEO dataset GSE33252). (**c**) Comparison of the correlation of uncorrected or corrected estimates with mRNA half-life measures. The Pearson correlation for Δ*exon*–Δ*intron* is 0.26, and for Δ*exon*–Δ*intron*–*bias* is 0.34. The error bars represent 95% confidence intervals. Note that REMBRANDTS filters against genes with low read counts (**Supplementary Figure 3a**), and therefore the set of genes used in this figure are a subset of those used in ref [3]. Large read counts are overall associated with larger bias terms (**Figure 1c,e**), resulting in a smaller correlation between Δ*exon*–Δ*intron* and mRNA half-life compared to values reported in ref [3].

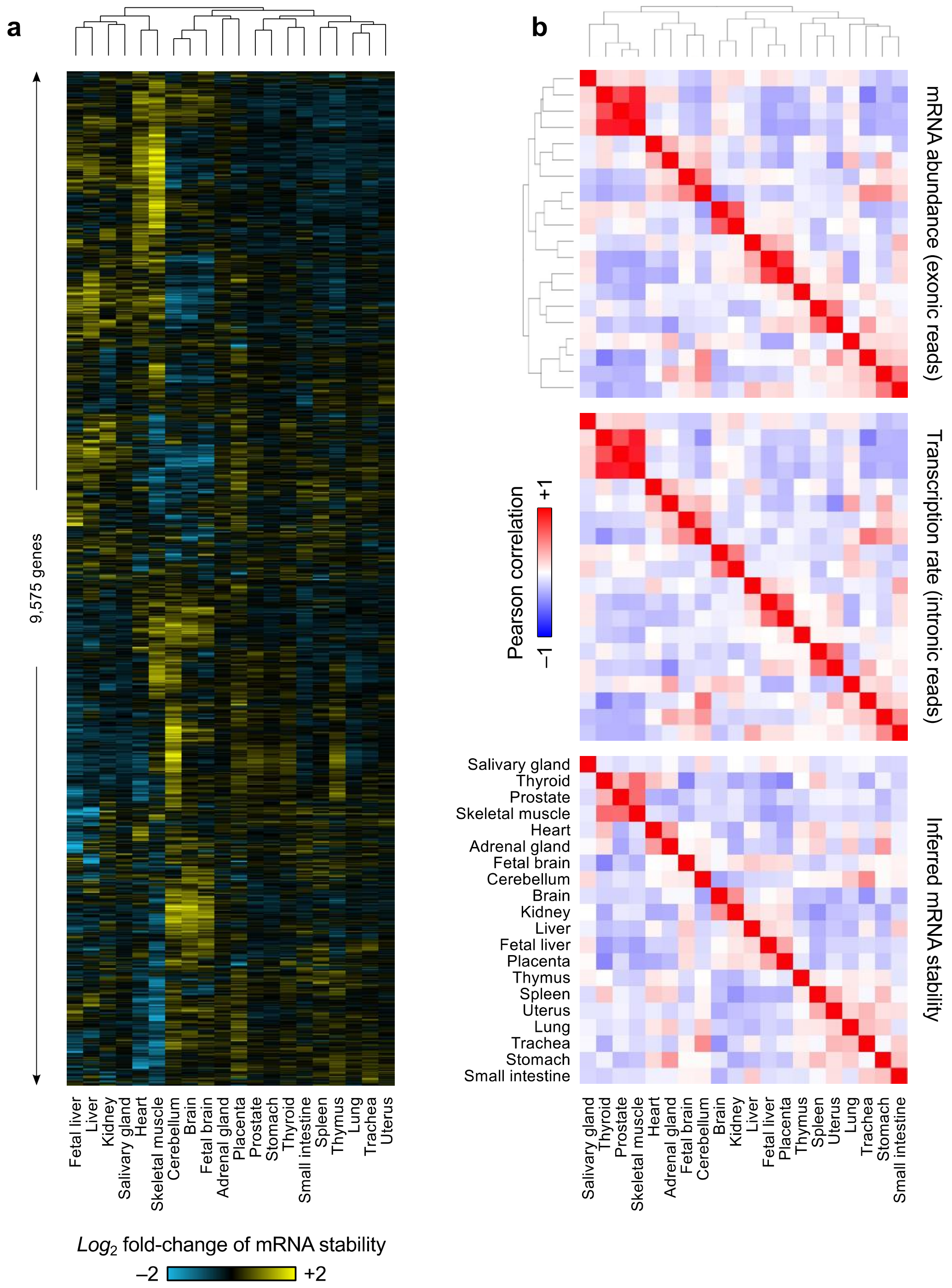

Supplementary Figure 6. Diversity of mRNA stability profiles across human tissues. (**a**) Heat map showing tissue-specific mRNA stability profiles. Each row represents one gene, and each column stands for one tissue. The color gradient represents the logarithm of fold-change of mRNA stability relative to average tissue. The dataset is available at http://csg.lab.mcgill.ca/sup/pan_stability/. (**b**) Similarity of mRNA abundance profiles (top), transcription rate profiles (middle), and mRNA stability profiles (bottom) among different tissues. Tissues are clustered based on data in the top panel.

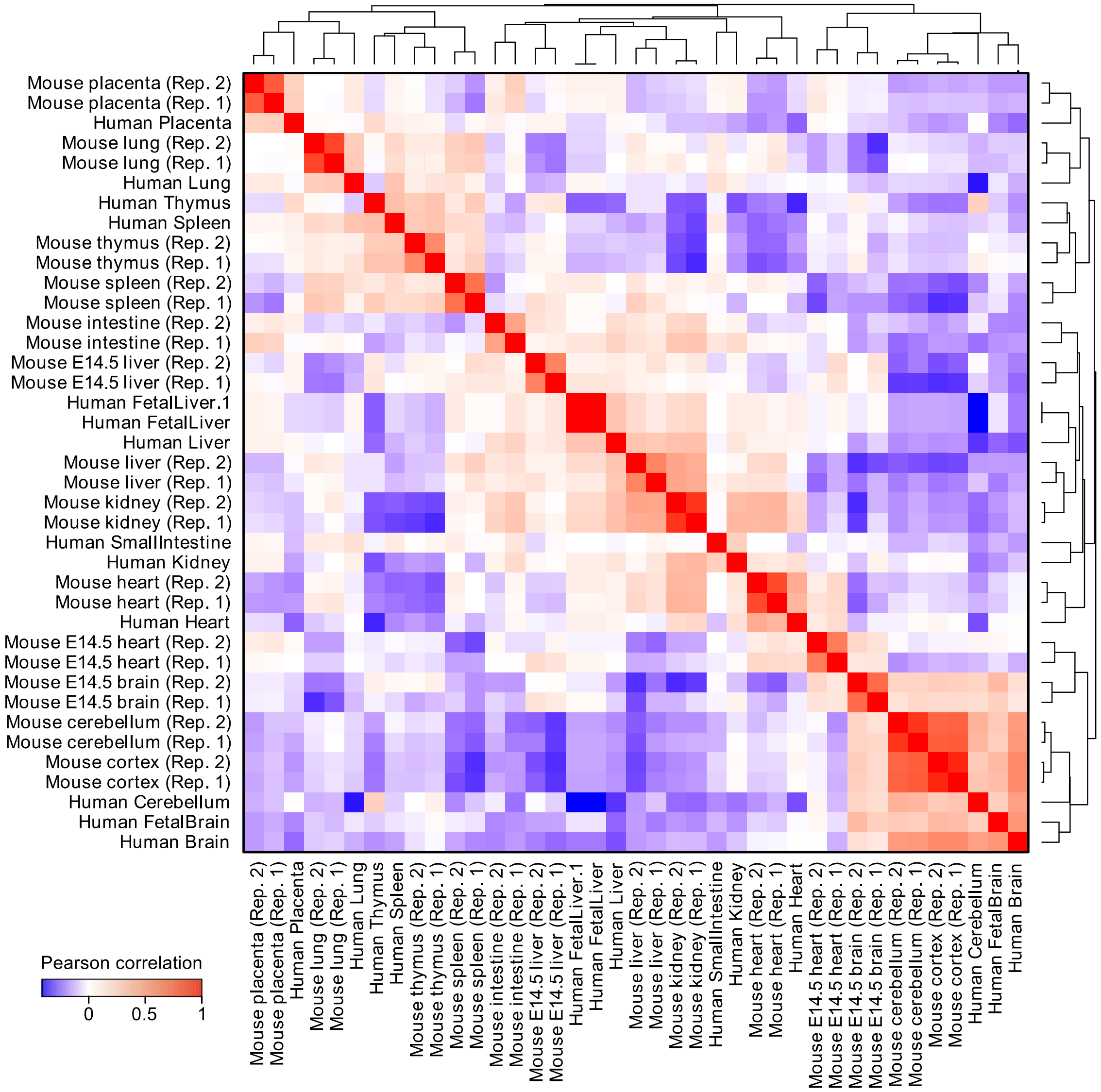

Supplementary Figure 7. Conservation of mRNA stability profiles between human and mouse tissues. The Mouse tissue-specific RNA-seq data were obtained from ref [7], and human RNA-seq data are from ref [4]. Only tissues that were present in both datasets are included in this figure.

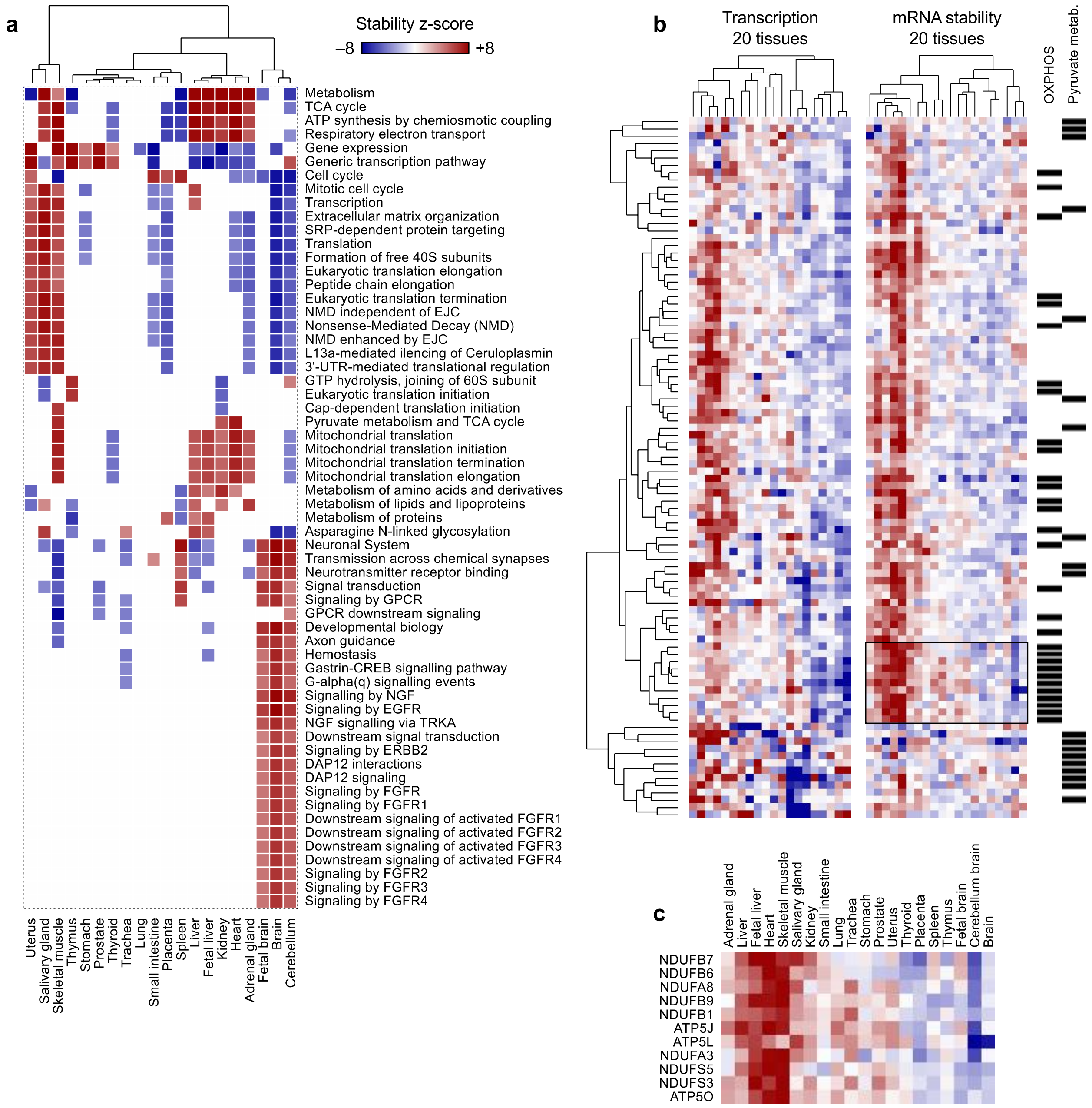

Supplementary Figure 8. Pathway enrichment analysis of tissue-specific mRNA stability measurements. (**a**) Top 60 REACTOME pathways with the most significant *P*-values for enrichment among stabilized (red) or destabilized (blue) genes. The color gradient corresponds to the Mann-Whitney U test z-score with respect to the bias-corrected measure of mRNA stability. The z-scores are shown only for pathway-tissue pairs that are significant at FDR<0.01. (**b**) The stability profiles of genes for an example pathway that shows differential regulation across multiple tissues (the citric acid cycle and respiratory electron transport, REACTOME R-HSA-1428517). Each row represents one gene, and each column corresponds to one tissue. The color gradient represents logarithm of fold-change of transcription (left) or stability (right) relative to average tissue, with red and blue indicating up- and down-regulation, respectively. The black bars on the right highlight genes that belong to the OXPHOS or pyruvate metabolism sub-pathways. The box in the mRNA stability heat map highlights a cluster of OXPHOS genes that are up-regulated only post-transcriptionally in metabolically active tissues, also shown in panel (**c**).

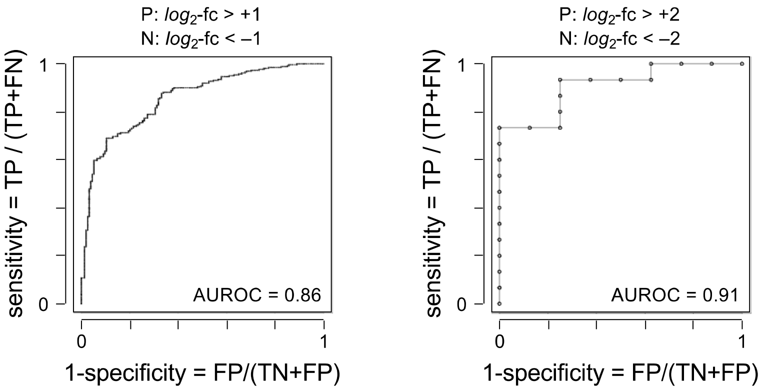

Supplementary Figure 9. Our brain mRNA stability model distinguishes brain-stabilized transcripts from brain-destabilized transcripts. The positive objects for classification were defined as genes that show >2-fold up-regulation (left panel) or >4-fold up-regulation (right panel) in both the Illumina BodyMap 2.0 dataset and the dataset by Duff et al. [4]. The negatives were defined as genes that are >2-fold down-regulated (left panel) or >4-fold down-regulated (right panel) in both datsets. The graphs represent receiver operating characteristic (ROC) curves for distinguishing positive objects (stabilized genes) from negative objects (destabilized genes), obtained by 10-fold cross-validation. TP: true positive; FP: false positive; TN: true negative; FN: false negative; AUROC: area under the ROC curve.

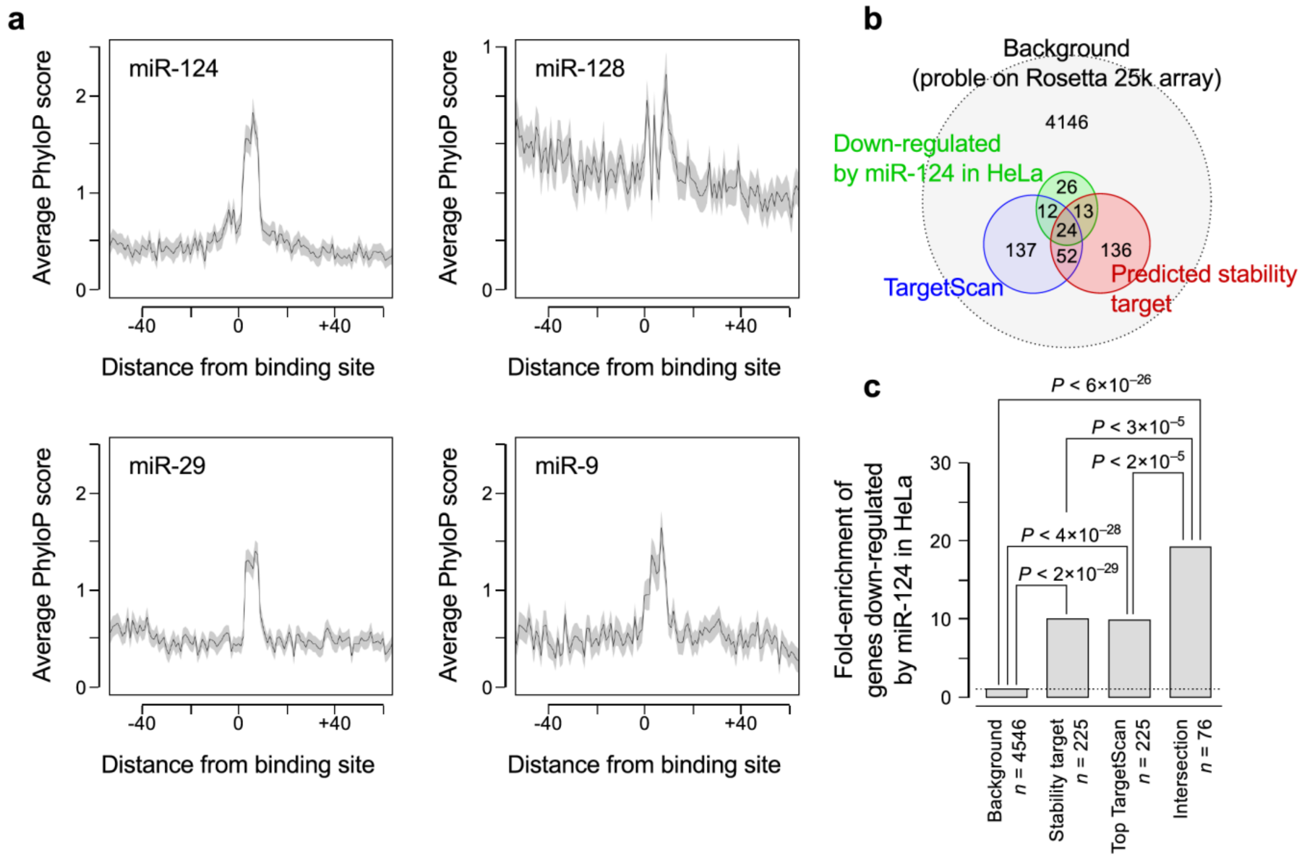

Supplementary Figure 10. Brain mRNA stability measurements and conservation-based approaches provide orthogonal information for identification of functional miR-124 targets. (**a**) The conservation profiles for high-confidence miRNA binding sites that were not previously reported in the literature (based on miRTarBase collection [8]). The shaded area represents the standard error of mean. (**b**) The Venn diagram of high-confidence stability targets of miR-124, miR-124 targets as predicted by TargetScan based on analysis of binding site conservation[9], and genes that are down-regulated after ectopic expression of miR-124 in HeLa cells[10]. For comparability, only the top 225 most conserved TargetScan targets are included (equal to the number of miR-124 targets in the high-confidence stability network). (**c**) Enrichment of genes that are down-regulated by miR-124 expression in HeLa cells[10] among high-confidence stability targets of miR-124, TargetScan predictions, and the intersection of the two sets. *P*-values are based on Fisher's exact test.

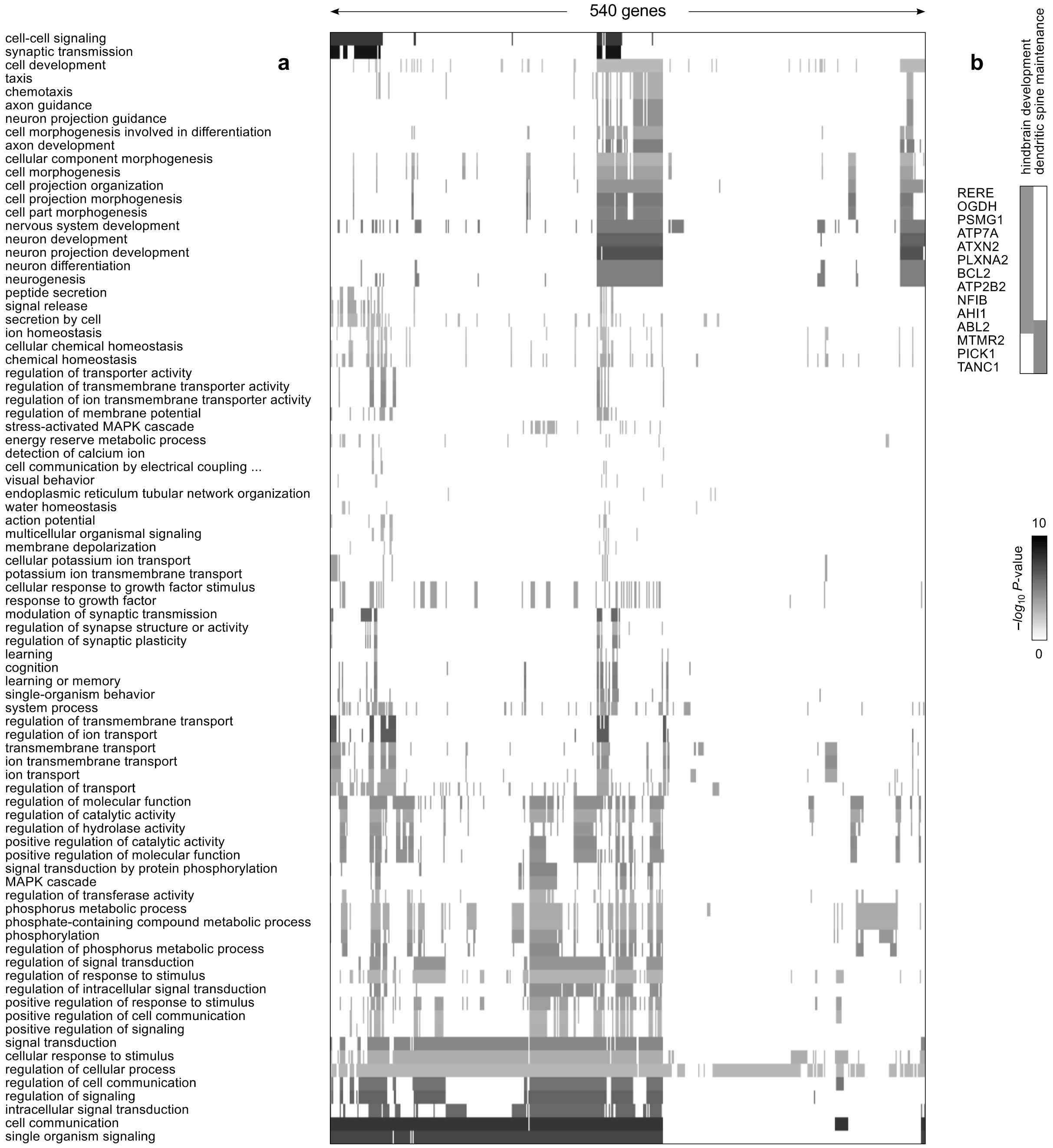

Supplementary Figure 11. Pathway enrichment among brain stability targets of RNA-binding proteins. (**a**) Biological processes that are enriched among targets of RBFOX (Fisher's exact test, FDR<0.05). Each row represents one Gene Ontology [11] biological process, and each column corresponds to one RBFOX target gene. The black/grey color in the heat map highlights gene-process associations. The color gradient represents the *P*-value of enrichment of the pathway among RBFOX stability targets (Fisher's exact test). Only RBFOX targets that belong to at least one of the significant biological processes are shown here. (**b**) Biological processes that are enriched among stability targets of ZFP36. Annotations are similar to (a).

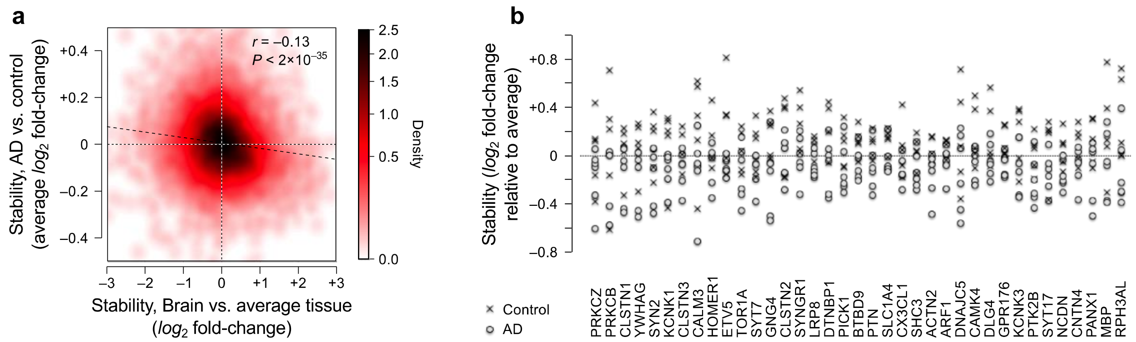

Supplementary Figure 12. De-regulation of mRNA stability in Alzheimer's disease (AD). (**a**) Scatter plot of the stability change in AD vs. the stability profile of brain. (**b**) Stability of synaptic transmission transcripts (GO:0007268) in the brain of AD individuals (circles) and control subjects (crosses). Out of the top 500 AD-destabilized genes, 37 genes (shown in this panel) belong to the synaptic transmission process, corresponding to ~2.4-fold enrichment of synaptic transmission genes among AD-destabilized transcripts (Fisher's exact test $P < 5\times10^{-7}$).

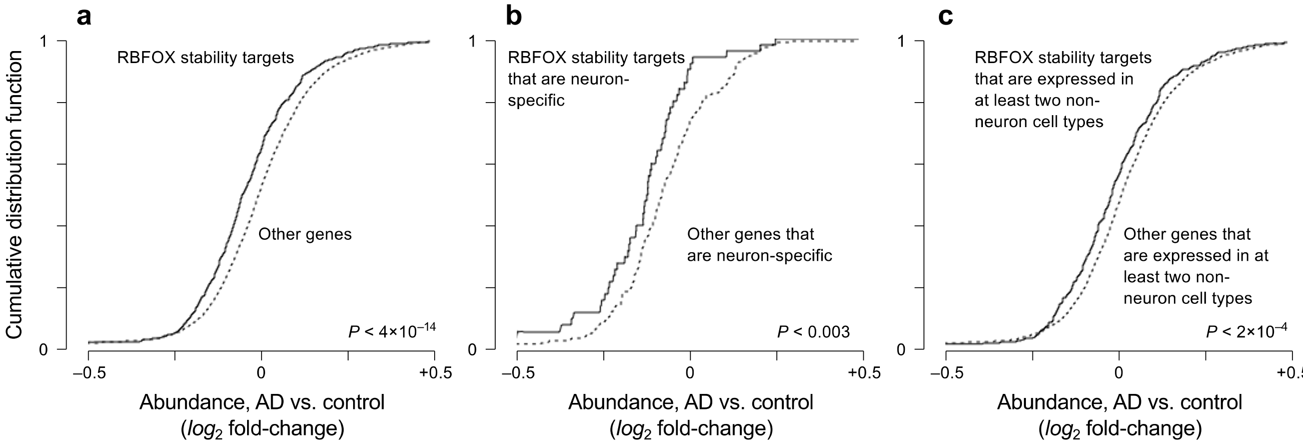

Supplementary Figure 13. Down-regulation of RBFOX targets in AD brain. Each graph shows the cumulative distribution of AD vs. control differential abundance for a background set of genes (dashed line) or stability targets of RBFOX proteins (solid line). (**a**) Analysis of all genes. (**b**) Analysis of neuron-specific genes, defined based on cell type-specific RNA-seq data from mouse cerebral cortex[12]. Neuron-specific genes were defined as genes that have >2-fold larger FPKM in neurons compared to the maximum FPKM in astrocytes, oligodendrocyte precursor cells, newly formed oligodendrocytes, myelinating oligodendrocytes, microglia, and endothelial cells. (**c**) Analysis of genes that are not specific to neurons, based on RNA-seq data from mouse cerebral cortex[12]. This plot includes only genes whose FPKM in at least two of the non-neuronal cell types is at least as large as that of neurons. For (b) and (c), mouse genes were mapped to human genes based on orthology assignments by Ensembl[13], including only one-to-one high-confidence orthologs. *P*-values correspond to Mann-Whitney U test.

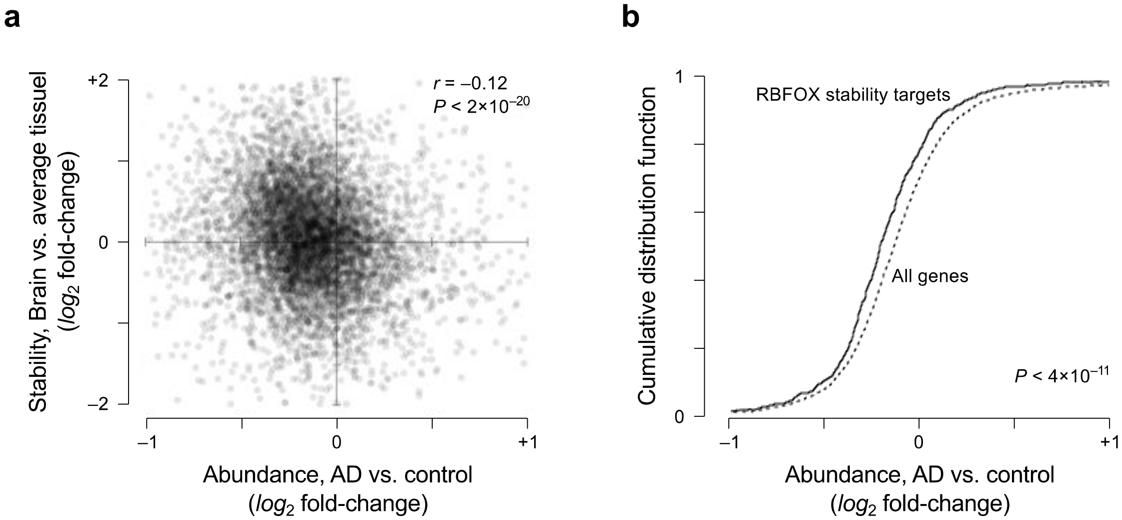

Supplementary Figure 14. mRNA abundance profiles in a validation cohort of AD individuals supports a role of mRNA stability and RBFOX network in AD. (**a**) Scatterplot of mRNA stability signature of brain (y-axis) vs. change in mRNA abundance in AD individuals (x-axis). AD (n=4) and control (n=4) brain RNA-seq data are from ref. [14]. (**b**) Cumulative distribution of AD vs. control differential abundance for all genes (dashed line) or stability targets of RBFOX proteins (solid line). *P*-value corresponds to Mann-Whitney U test.

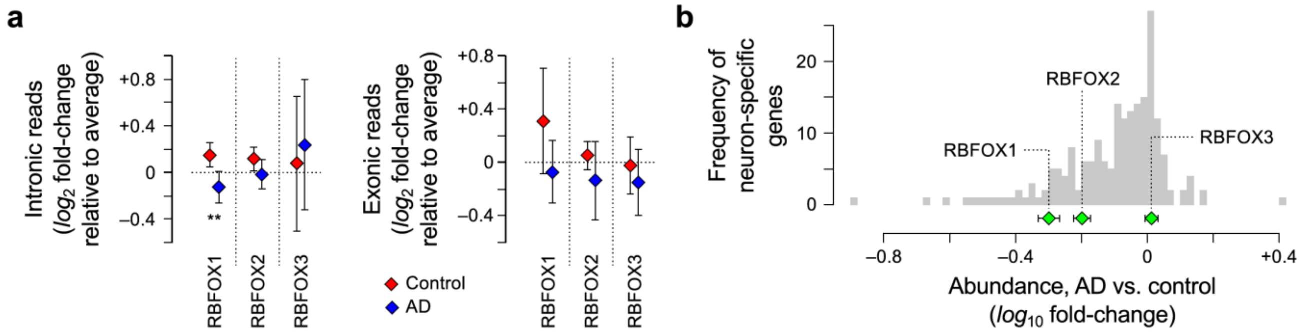

Supplementary Figure 15. Transcription rate and abundance of RBFOX genes in the brain of AD and control individuals. (**a**) Intronic and exonic fragment abundance of RBFOX1/2/3 genes based on RNA-seq data from ref [15]. The error bars represent the standard error. (**) P<0.01, two-sided Student's t-test. (**b**) Differential abundance of RBFOX1/2/3 genes in AD (n=310) vs. control individuals (n=157), based on microarray data from ref [16]. The error bars represent standard error of the mean. The histogram represents the differential abundance of 201 highly neuron-specific genes (FPKM in neuron >10-fold higher than the maximum FPKM in astrocytes, oligodendrocyte precursor cells, newly formed oligodendrocytes, myelinating oligodendrocytes, microglia, and endothelial cells in mouse cerebral cortex[12]).

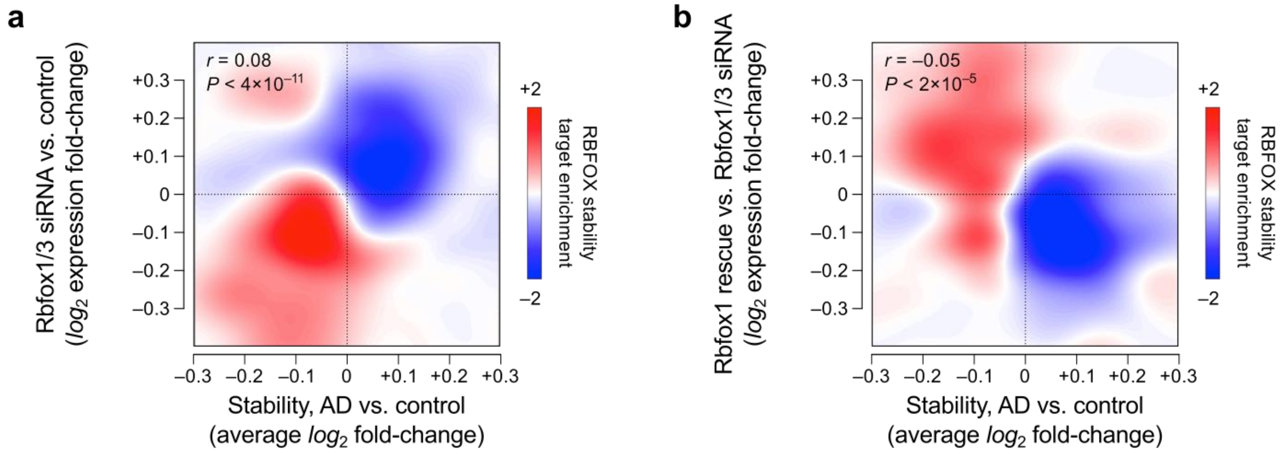

Supplementary Figure 16. Mouse Rbfox1 regulates the abundance of orthologs of AD-destabilized RBFOX targets. (**a**) Enrichment of RBFOX targets among transcripts that are destabilized in AD and whose orthologs are down-regulated after knock-down of Rbfox1/3 in mouse [17]. The color gradient represents the density of genes that are RBFOX stability targets minus density of other genes (red: higher density of RBFOX targets). (**b**) Enrichment of RBFOX targets among transcripts that are rescued by ectopic expression of a cytoplasmic form of Rbfox1 in cells harboring siRbfox1/3. Data from ref. [17].

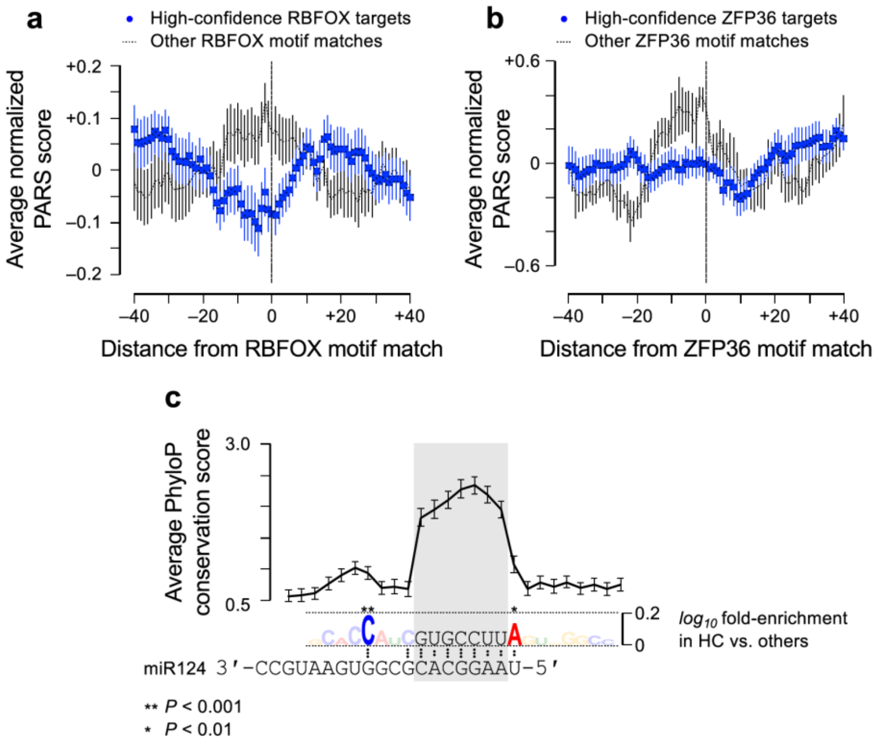

Supplementary Figure 17. Structur and sequence context at high-confidence binding sites of RBFOX, ZFP36, and miR-124. (**a**) PARS score profile[18] around RBFOX motif matches for high-confidence RBFOX targets and other RBFOX motif-containing transcripts. To calculate the genome-wide PARS scores, renatured S1 and V1 data for cell lines GM12878, GM12891, and GM12892 were obtained from GEO entry GSE50676. Reads from different cells were pooled together and were mapped to GRCh38 genome assembly using HISAT2[19], and PARS scores were calculated as previously described [18]. Binding sites with fewer than 100 read ends mapping to their ±100bp neighborhood were filtered. Larger PARS scores correspond to higher likelihood of participating in double-stranded RNA. The error bars represent standard error of mean. (**b**) PARS score profile around ZFP36 binding sites. (**c**) The sequence context around miR-124 binding sites. At the bottom, the height of the colored bases represents their fold-enriched in high-confidence targets relative to other sequences with a match to the miR-124 seed sequence. The miR-124 seed sequence itself is highlighted with the grey box. Bases that are significantly more abundant in high-confidence targets compared to other seed matches are highlighted (** P<0.005; * P<0.02). The upper graph shows the PhyloP conservation score profile for high-confidence targets that match the most abundant bases at the two significant positions (i.e C and A, respectively). The error bars represent standard error of mean.

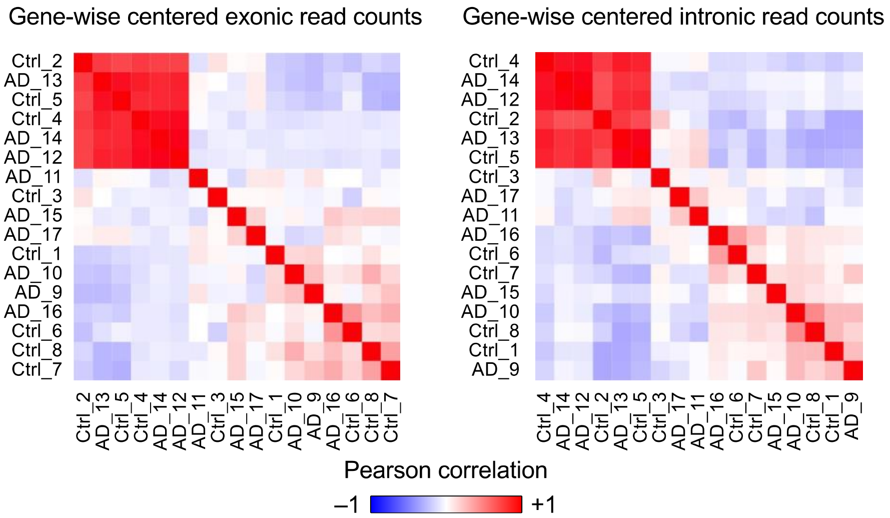

Supplementary Figure 18. Clustering of RNA-seq data of AD and control subjects from ref [15]. The panels show the clustering of samples based on Δ*exon* (left) or Δ*intron* (right) values of all genes. Samples Ctrl_2, AD_13, Ctrl_5, Ctrl_4, AD_14 and AD_12 were removed from analysis due to unusually strong clustering, which stemmed from a large number of zero-read genes.

Supplementary Table 1. Primer sequences for qRT-PCR experiments.

RefSeq Accession	Gene name	Forward (5' → 3')	Reverse (5' → 3')	Product length
NM_001025593	ARFIP1	AGTGCCAGGATTGAATATGATGC	TTTGGCAGAGTGTTTGCGTC	83
NM_001040157	CEP44	AGAGGTGGACTGTGTAGGTTTG	AAAAGAATAGCTGATGATGGGCA	70
NM_001039469	MARK2	CCTGACTGGGAAAGAGGTAGCTG	TTTCTGGAGGCTGGAGGAGTTC	70
NM_001030006	AP2B1	AGGATGTTAGTTCTCTCTTTCCAGA	GGCTGACTCTTGGCGTAGTT	109
NM_024953	NAA25	TTATGCAATCTATATCGGCACAGGA	ATTCTCTCAGCAAGGGGCAG	70
NM_006451	PAIP1	TTCCAGTTACACAGAATCCTATGAG	ACAACCATTCAGGGTCTCTGC	148

SUPPLEMENTARY REFERENCES

1 Bousquet-Antonelli, C., Presutti, C. & Tollervey, D. Identification of a regulated pathway for nuclear pre-mRNA turnover. *Cell* **102**, 765-775 (2000).

2 Fabregat, A. *et al.* The Reactome pathway Knowledgebase. *Nucleic Acids Res* **44**, D481-487, doi:10.1093/nar/gkv1351 (2016).

3 Gaidatzis, D., Burger, L. & Stadler, M. B. Analysis of intronic and exonic reads in RNA-seq data characterizes transcriptional and post-transcriptional regulation. *Nat Biotechnol* **33**, 722-729, doi:10.1038/nbt.3269 (2015).

4 Duff, M. O. *et al.* Genome-wide identification of zero nucleotide recursive splicing in Drosophila. *Nature* **521**, 376-379, doi:10.1038/nature14475 (2015).

5 Furlow, P. W. *et al.* Mechanosensitive pannexin-1 channels mediate microvascular metastatic cell survival. *Nat Cell Biol* **17**, 943-952, doi:10.1038/ncb3194 (2015).

6 Goodarzi, H. *et al.* Metastasis-suppressor transcript destabilization through TARBP2 binding of mRNA hairpins. *Nature* **513**, 256-260, doi:10.1038/nature13466 (2014).

7 Shen, Y. *et al.* A map of the cis-regulatory sequences in the mouse genome. *Nature* **488**, 116-120, doi:10.1038/nature11243 (2012).

8 Chou, C. H. *et al.* miRTarBase 2016: updates to the experimentally validated miRNA-target interactions database. *Nucleic Acids Res* **44**, D239-247, doi:10.1093/nar/gkv1258 (2016).

9 Agarwal, V., Bell, G. W., Nam, J. W. & Bartel, D. P. Predicting effective microRNA target sites in mammalian mRNAs. *Elife* **4**, doi:10.7554/eLife.05005 (2015).

10 Lim, L. P. *et al.* Microarray analysis shows that some microRNAs downregulate large numbers of target mRNAs. *Nature* **433**, 769-773, doi:10.1038/nature03315 (2005).

11 Ashburner, M. *et al.* Gene ontology: tool for the unification of biology. The Gene Ontology Consortium. *Nat Genet* **25**, 25-29, doi:10.1038/75556 (2000).

12 Zhang, Y. *et al.* An RNA-sequencing transcriptome and splicing database of glia, neurons, and vascular cells of the cerebral cortex. *J Neurosci* **34**, 11929-11947, doi:10.1523/JNEUROSCI.1860-14.2014 (2014).

13 Yates, A. *et al.* Ensembl 2016. *Nucleic Acids Res* **44**, D710-716, doi:10.1093/nar/gkv1157 (2016).

14 Magistri, M., Velmeshev, D., Makhmutova, M. & Faghihi, M. A. Transcriptomics Profiling of Alzheimer's Disease Reveal Neurovascular Defects, Altered Amyloid-beta Homeostasis, and Deregulated Expression of Long Noncoding RNAs. *J Alzheimers Dis* **48**, 647-665, doi:10.3233/JAD-150398 (2015).

15 Scheckel, C. *et al.* Regulatory consequences of neuronal ELAV-like protein binding to coding and non-coding RNAs in human brain. *Elife* **5**, doi:10.7554/eLife.10421 (2016).

16 Narayanan, M. *et al.* Common dysregulation network in the human prefrontal cortex underlies two neurodegenerative diseases. *Mol Syst Biol* **10**, 743, doi:10.15252/msb.20145304 (2014).

17 Lee, J. A. *et al.* Cytoplasmic Rbfox1 Regulates the Expression of Synaptic and Autism-Related Genes. *Neuron* **89**, 113-128, doi:10.1016/j.neuron.2015.11.025 (2016).

18 Wan, Y. *et al.* Landscape and variation of RNA secondary structure across the human transcriptome. *Nature* **505**, 706-709, doi:10.1038/nature12946 (2014).

19 Kim, D., Langmead, B. & Salzberg, S. L. HISAT: a fast spliced aligner with low memory requirements. *Nat Methods* **12**, 357-360, doi:10.1038/nmeth.3317 (2015).
